# Editorial: Fascia as a multi-purpose structure of connective tissue - dysfunction, diagnostics and treatment

**DOI:** 10.3389/fmed.2024.1472116

**Published:** 2024-08-14

**Authors:** Anna Mika, Łukasz Oleksy, Caterina Fede, Carmelo Pirri, Carla Stecco

**Affiliations:** ^1^Institute of Clinical Rehabilitation, University of Physical Education in Krakow, Kraków, Poland; ^2^Faculty of Health Sciences, Department of Physiotherapy, Jagiellonian University Medical College Krakow, Kraków, Poland; ^3^Department of Orthopedics, Traumatology and Hand Surgery, Faculty of Medicine, Wrocław Medical University, Wrocław, Poland; ^4^Oleksy Medical & Sports Sciences, Rzeszów, Poland; ^5^Department of Neurosciences, Institute of Human Anatomy, University of Padua, Padua, Italy

**Keywords:** fascia, musculoskeletal system, diagnostic methods, rehabilitation, treatment, fascial dysfunction

For many years, the fasciae have been considered only as a “white envelope for the muscles”, and very little attention has been given to their macroscopic and histological anatomy. With the last researches it is clear that we can recognized many fasciae, each one with specific features: the superficial fascia, the muscular fasciae, the visceral fasciae and the neural fasciae. The superficial fascia is rich in elastic fibers (1), it is adaptable and strongly connected with the skin (2), it envelops and protect the superficial vessels and nerves and probably plays a key role in lymphatic drainage (3), and in tissue healing and regeneration processes (4). The muscular (or deep) fasciae are distinguished in two big groups: the aponeurotic fasciae, that work as a bridge connecting different muscles, and the epimysial fasciae, specific for each muscle. The deep fasciae are formed by collagen fibers organized in layers, and each layer is separated by the closer one by loose connective tissue, rich in water and hyaluronan (HA) (5). The collagen fibers define the mechanical behavior of fasciae, the hyaluronan defines the tissue hydration and the ability of glide. All these elements could be altered by trauma, bad posture, immobilization. The visceral fasciae envelope the viscera and define their mobility and motility (6). They have a rich autonomic innervation and connect the organs to the locomotor system in very precise points. Finally, also the meningeal layers could be considered specialized fasciae.

The fasciae are very well-innerved (more than muscles, tendons, and joints), both with sensitive an autonomic innervation, so much to be considered a sensory organ (7). We can distinguished three different type of innervation inside fasciae:

- Free nerve ending forming a network, totally embedded in the fasciae, and able to perceive every change in the fascial tension. These receptors have a key role in proprioception and in the perception of the motor directions, but also they could be able to perceive pain (8).
- Autonomic fibers, they are around 35% in the superficial fascia, a little less in the deep fasciae, much more in the visceral ones. They are present above all around the main vessels, and consequently they are responsible of the regulation of the blood flow inside the fasciae, but also in the middle of the connective tissue, and consequently they could be involved in the fibrotic process of fascial tissue (9).
- The muscle spindles. They are a specific innervation of the epimysial fasciae. Indeed these corpuscles are totally embedded in the perimysium, and their capsule is nothing more than a doubling of the perimysium around the intrafusal fibers. Muscle spindles inform the Central Nervous System (CNS) of the continually changing status of muscle tone, movement, loss of normal elasticity, position of body parts, absolute length of muscle and rate of change (velocity) of the length of the muscle. In order for a muscle spindle to function it must be able to lengthen, shorten and glide to allow its annulospiral and flower spray organs to be stretched to report accurate information to the CNS (10), and this is made possible by the epimysial fascia.

So, fasciae could be considered a key element in peripheral motor coordination and proprioception, but also of interoception. At the same time, altered (restricted, densified) fasciae are responsible for chronic stiffness, decreased strength and abnormal movement patterns (loss of motor direction of bodily segments), bad motility of the internal organs, altered lymphatic drainage (11).

**Figure 1:**
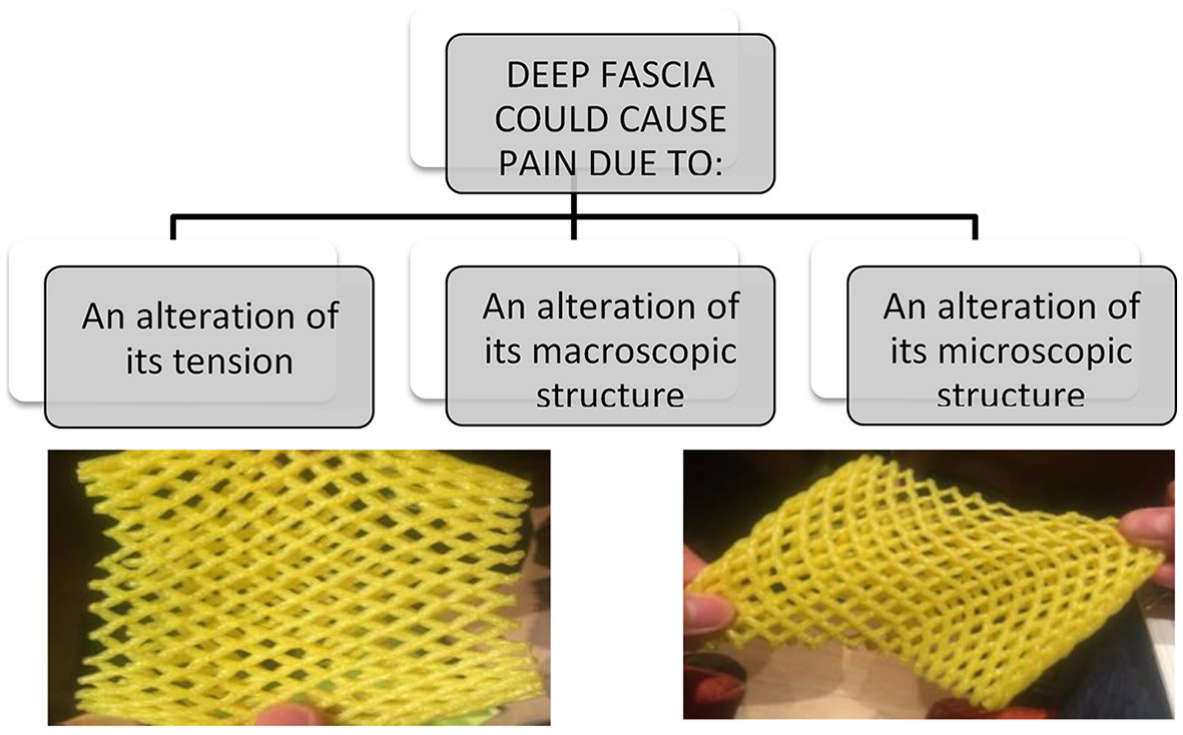
Fascia could cause pain in different modalities, and they required different types of treatment and different way to make diagnosis. One of the most common cause of fascial pain is an alteration of the fascial tension, that cause a deformation of the fascial free nerve ending.

Besides, the fasciae could be subjected to different types of alterations, some are clearly visible with standard imaging evaluations, other need a biopsy to be understood. In a schematic way, we can distinguish three different conditions (Figure 1):

- The fascia has only an anomalous tension, due to the overstretching due to the underlying muscles that insert into it or to postural alteration. In this case the fascia is healthy, but the nerve receptors inside are constantly triggered. Wilke et al. (12) demonstrated the strain transfer along fasciae. In particular, the Authors checked the range of motion of the neck in 26 healthy participants before and after the stretching of the inferior limbs. After stretching, the cervical Range of Motion (ROM) was improved, demonstrating the anatomical continuity of the fasciae of inferior limbs and neck. From a clinical point of view, it means that a trauma or a tension in the inferior limbs can affect the neck mobility, and consequently some patients maybe don't answer to our treatments because they are addressed at the wrong area. Further, it means that it is important to assess in a global way a patient with myofascial pain, because the fascial alteration could be far away from the site of pain.
- The fascia has macroscopic alterations, such as a scar after surgery or trauma. These alterations could be seen with the standard ultrasound evaluation, but also with Magnetic Resonance Imaging (MRI) and TC. Stecco et al. (13) evaluated 25 subjects with chronic ankle instability with MRI, demonstrating in 21 patients specific alterations of the ankle retinacula (that are fascial thickenings), such as edema, interruption of continuity, thickening or adhesion to the subcutaneous layers. These alterations were observed separately or in association. In a recent review, Pirri et al. (14) listed all the Ultrasound (US) parameters that can be altered in the fasciae and that can allow to do a diagnosis of fascial pathology: thickness, echogenicity, stiffness, deformation, shear strain, and displacement.
- The fascia can be altered in their molecular components, such as the amount of collagen and elastic fibers, or of hyaluronan, or in the density of innervation and vascularization. In this case, the imaging evaluations show a standard macroscopic organization of the fasciae, and only the biopsy can highlight the alteration. In the last years Fede et al. (15) demonstrated as the quantity of elastic and collagen fibers vary with aging, as happens in all the subcutaneous tissues. It was recently demonstrated that different levels of estrogenic hormones can modulate the production of collagen I, collagen III and fibrillin (16). It seems that post-menopausal fasciae have more collagen type I (8.4 vs. 5.2% in control) and less collagen III (1.5 vs. 2.4%), explaining why the fasciae become more rigid with aging. On the contrary, fasciae become more elastic during pregnancy, with increased amounts of collagen-III (6.7%) and fibrillin (3.6%, compared to 0.5% of the control) and a corresponding decrease of collagen I (1.9%). Mechanical inputs, such as extracorporeal shock wave, also seems to change the fiber composition in fasciae, activating gene expression for transforming growth factor β1 and collagen types I and III (17, 18). With diabetes there are chronic alterations of the connective tissue, with a thickening of the collagen fibers and a fragmentation of the elastic fibers, leading to fascial stiffness (19, 20). The synthesis of the collagen type III fibers increases in diabetic subjects, whereas the synthesis of collagen type I fibers decreases. Furthermore, Extracellular Matrix (ECM) turnover in diabetes patients is affected by chronic hyperglycemia, determining the accumulation of larger quantities of collagen, resulting in ECM thickening. Fantoni et al. observed (21) a relationship between fascial pathology and hip osteoarthritis (OA): in OA patients Authors demonstrated an increase in Collagen I (COL I), along with the reduction of Collagen III (COL III) and HA, leading to fascial stiffening, which could alter fascial mechanics and be linked to the development and symptoms of OA.

Another element that can alter the microanatomy of fasciae are the myofibroblasts. According to Schleip et al. (22), myofibroblasts are present in all fasciae, but cell density increases in many pathological conditions, such as palmar fibromatosis, Morbus Ledderhose, hypertrophic scars, and similar fascial fibrotic conditions, but also in chronic low back pain, where myofibroblasts are associated with an augmented occurrence of (micro-) injuries and related cellular repair processes. It seems that fascial alteration could be a trigger for the transformation of fibroblasts into myofibroblasts but, due to their contractile activity, myofibroblasts further increase fascial tension creating a loop that progressively aggravates the problem. Fasciae express also cannabinoid receptors [both Cannabinoid 1 and 2 (CB1 and CB2)] and their stimulation seems to induces the production of hyaluronan-rich vesicles, leading to greater tissue fluidity (23, 24).

To conclude, we think that it is time that fasciae will be considered in the clinical setting because there are many evidences that they can be a source of pain. The various aspects of diagnosing fascia disorders and the effectiveness of the applied therapy methods have been confirmed in studies published in this Research Topic, for the treatment of allergies (Liu et al.), joint pain and joint disorders (Liu and Wang; Rogers et al.) and the most common back pain (Brandl et al.), highlighting that the fascia should be considered for diagnosis and treatment in heterogeneous and diverse clinical pictures. However, it is important to consider that, to understand the fascial alteration and how improve a fascial alteration, it is important to consider that fasciae form a three dimensional network and consequently the point at which the patient feels pain often does not correspond at the origin of the fascial problem. This implies that if we evaluate with ultrasound, MRI or other instruments the fascia where the patient feels pain, probably we are not able to see any alteration, but this does not automatically mean that this is not a fascial problem. Besides, the assessment of a patient with fascial problems required always a global analysis, considering previous trauma and previous unbalance conditions of the fasciae.
